# Novel angiotensin-converting enzyme inhibitory peptides from tuna byproducts—milts: Preparation, characterization, molecular docking study, and antioxidant function on H_2_O_2_-damaged human umbilical vein endothelial cells

**DOI:** 10.3389/fnut.2022.957778

**Published:** 2022-07-22

**Authors:** Shi-Kun Suo, Shuo-Lei Zheng, Chang-Feng Chi, Hong-Yu Luo, Bin Wang

**Affiliations:** ^1^Key Laboratory of Health Risk Factors for Seafood of Zhejiang Province, College of Food Science and Pharmacy, Zhejiang Ocean University, Zhoushan, China; ^2^National and Provincial Joint Laboratory of Exploration and Utilization of Marine Aquatic Genetic Resources, National Engineering Research Center of Marine Facilities Aquaculture, School of Marine Science and Technology, Zhejiang Ocean University, Zhoushan, China

**Keywords:** skipjack tuna (*Katsuwonus pelamis*), milt, peptide, antihypertensive function, angiotensin-I-converting enzyme (ACE), antioxidant activity

## Abstract

To prepare peptides with high angiotensin-converting enzyme (ACE) inhibitory (ACEi) activity, Alcalase was screened from five proteases and employed to prepare protein hydrolysate (TMH) of skipjack tuna (*Katsuwonus pelamis*) milts. Subsequently, 10 novel ACEi peptides were isolated from the high-ACEi activity TMH and identified as Tyr-Asp-Asp (YDD), Thr-Arg-Glu (TRE), Arg-Asp-Tyr (RDY), Thr-Glu-Arg-Met (TERM), Asp-Arg-Arg-Tyr-Gly (DRRYG), Ile-Cys-Tyr (ICY), Leu-Ser-Phe-Arg (LSFR), Gly-Val-Arg-Phe (GVRF), Lys-Leu-Tyr-Ala-Leu-Phe (KLYALF), and Ile-Tyr-Ser-Pro (IYSP) with molecular weights of 411.35, 404.41, 452.45, 535.60, 665.69, 397.48, 521.61, 477.55, 753.91, and 478.53 Da, respectively. Among them, the IC_50_ values of ICY, LSFR, and IYSP on ACE were 0.48, 0.59, and 0.76 mg/mL, respectively. The significant ACEi activity of ICY, LSFR, and IYSP with affinities of −7.0, −8.5, and −8.3 kcal/mol mainly attributed to effectively combining with the ACEi active sites through hydrogen bonding, electrostatic force, and hydrophobic interaction. Moreover, ICY, LSFR, and IYSP could positively influence the production of nitric oxide (NO) and endothelin-1 (ET-1) secretion in human umbilical vein endothelial cells (HUVECs) and weaken the adverse impact of norepinephrine (NE) on the production of NO and ET-1. In addition, ICY, LSFR, and IYSP could provide significant protection to HUVECs against H_2_O_2_ damage by increasing antioxidase levels to decrease the contents of reactive oxide species and malondialdehyde. Therefore, the ACEi peptides of ICY, LSFR, and IYSP are beneficial functional molecules for healthy foods against hypertension and cardiovascular diseases.

## Introduction

Hypertension, also known as high blood pressure, is the most critical factor influencing the morbidity and mortality of cardiovascular disease (CVD) and renal disease (1–3). The prevalence of hypertension is about 1.3 billion, and this population may grow to 1.56 billion by 2030, which will cause a global economic burden of $274 billion (4). Oral medication is the conventional therapeutic intervention in hypertension management, and finding new drug is the priority to effectively control and manage the hypertensive population (5, 6). Angiotensin-converting enzyme (ACE) plays a vital physiological function in controlling blood pressure by converting angiotensin (Ang) I to Ang II by deactivating the vasodilator bradykinin (7, 8). In consequence, the synthetic ACE inhibitors [captopril (Cap), lisinopril, enalapril, etc.] have been used to curing hypertension, diabetic nephropathy, stabilization of antioxidant responses, and endothelial dysfunction (6, 7). Unfortunately, commercially available ACE inhibitors are like any other synthetic drugs and present many serious side effects that need to be careful in their prescription administration (6, 9, 10). Therefore, it has become an inevitable trend to find safer, affordable, and effective ACEis from natural resources to replace synthetic drugs for the treatment of hypertension and CVD.

Currently, some natural ACE inhibitors, including peptides, flavones, terpenoids, alkaloids, and steroids, have been purified from different plants, animals, and microorganisms (6, 11–13). Among them, ACEi peptides have attracted wide interest due to their high nutritional value and significant biological activity (14–19). In addition, global fish production reached around 179 million tons, and approximately 50% of catches become byproducts during factory processing (20–23). Those fish byproducts result in burdensome disposal problems, and unreasonable treatment will give rise to serious environmental pollution (24–26). For making full use of these fish byproducts, many ACEi peptides were prepared from different processing byproducts, such as tuna bone (27, 28), skate (*Okamejei kenojei*) skin (29), mackerel skin (30), Nile tilapia skin (31) and skeleton (32), sea cucumber (*Stichopus japonicus*) gonad (33), smooth-hound (*Mustelus mustelus*) viscera (34), Atlantic salmon skin (35), Alaska pollack skins (36), and rainbow trout (*Oncorhynchus mykiss*) viscera (37). Those sea-food derived ACEi peptides exhibit high potential application value in terms of diet and clinical therapeutics on anti-hypertension (5, 38, 39).

Tuna is one of the world's foremost commercial deep-sea fish, with about 7.9 × 10^6^ tons of catches in 2018, and it is crucial for the balanced nutrition and optimal health because it provides a variety of high-quality nutritional and functional ingredients (23, 40, 41). Skipjack tuna (*Katsuwonus pelamis*) is the most productive and low-value species of tuna, with catches of 3.2 × 10^6^ tons (23). In the manufacturing process of canned fish, about half tuna materials are taken for byproducts (42–46). To make the most of these byproducts, bioactive peptides were produced from tuna byproduct proteins, such as dark muscles (47–49), bone/frame (25, 27), scale (50), roe (45, 51), and head and viscera (52, 53). In our previous study, 13 antioxidant oligopeptides were prepared and identified from the Neutrase hydrolysate of skipjack tuna milts (46). Among them, SMDV, SVTEV, PHPR, VRDQY, and GHHAAA presented significant cytoprotection on H_2_O_2_-damaged human umbilical vein endothelial cells (HUVECs) (46). Furthermore, to make more efficient use of this resource, the objectives of this research were to isolate, identify, and evaluate the activity of ACEi peptides from protein hydrolysate of skipjack tuna milts. Moreover, the ACEi mechanism of isolated peptides was illustrated by the molecular docking experiment.

## Materials and methods

### Materials

Skipjack tuna milts were provided by Ningbo Today Food Co., Ltd (China). The Nitric Oxide (NO) Assay Kit (A012-1) and Endothelin-1 (ET-1) ELISA Kit (HM10108) were bought from Nanjing Jiancheng Bioengineering Institute (China). Alcalase (CAS No.: 9014-01-1), glutathione (GSH) (CAS No.: 70-18-8), trypsin (CAS No.: 9002-07-7), trifluoroacetic acid (TFA) (CAS No.: 76-05-1), N-[3-(2-furyl)acryloyl]-Phe-Gly-Gly (FAPGG) (CAS No.: 64967-39-1), 2-[4-(hydroxyethyl)-1-piperazinyl]ethanesulfonic acid (HEPES) (CAS No.: 7365-45-9), pepsin (CAS No.: 9001-75-6), papain (CAS No.: 9001-73-4), Cap (CAS No.: 62571-86-2), and ACE (CAS No.: 9015-82-1) were purchased from Sigma-Aldrich (Shanghai) Trading Co., Ltd. (China). Norepinephrine (NE) (CAS No.: 51-41-2), 3-(4,5-dimethylthiazol-2-yl)-2,5-diphenyltetrazolium bromide (MTT) (CAS No.: 298-93-1), and Neutrase (CAS No.: 9068-59-1) were bought from Beijing Solarbio Science & Technology Co., Ltd (China). Peptides of TP1–TP10 (purity > 95%) were synthesized in Shanghai Peptide Co. Ltd. (China).

### Determination of ACEi activity

The ACEi activity was tested using the method described by Zhao et al. (8). In brief, 50 μL FAPGG solution as a substrate (1 mM) in HEPES-HCl buffer (0.5 mM, pH 8.3, containing 300 mM salt) was mixed with 40 μL sample (5, 10, 20, 40 mg/mL) and 10 μL of ACE solution. The mixture was pre-incubated at 37°C for 5 min. Then, 50 μL of 1.0 mol/L FPAGG solution was added to the mixture to initiate the reaction and incubated at 37°C for 30 min. The control was prepared using 80 mM HEPES-HCl buffer containing 300 mM NaCl (pH 8.3), instead of the sample. The sample group and control group were run in the same manner. After that, the absorbance of the sample solution was measured at 340 nm. All samples were measured as described before, respectively. The IC_50_ value was defined as the concentration of inhibitor required to inhibit 50% of the ACE activity. The ACEi activity was calculated by using the following equation:


$$\begin{array}{l} \text{ACEi activity}\left(\text{\%}\right)=\left(1-B_{0}-B_{30}/A_{0}-A_{30}\right)\times \;100 \end{array}$$

A_0_ and B_0_ represent the initial absorbance of the control group and the sample group; A_30_ and B_30_ represent the absorbance after 30 min for the control group and the sample group.

### Preparation of protein hydrolysate of tuna milts

Tuna milts were degreased using isopropanol as per the reported method (46). In brief, isopropanol was added to the milt homogenate with a liquid/solid ratio of 4:1 (v/w), and the mixed solution was homogenized and kept at 20 ± 2°C for 60 min. Afterward, the mixed solution was centrifuged at 6,000 rpm for 0.5 h, and the resulted residue was defatted at room temperature for 1.5 h using isopropanol with a liquid/solid ratio of 4:1 (v/w). Finally, the resulted residue was dried at 35 ± 2°C. After that, the defatted milt powders were dispersed in buffered solution (0.2 M, w/v) and separately hydrolyzed using Alcalase (55°C, pH 9.5), trypsin (37.5°C, pH 7.8), pepsin (37.5°C, pH 2.0), papain (55°C, pH 7.0), and Neutrase (55°C, pH 7.0), respectively, with enzyme dose of 2% (w/w). According to the designed hydrolysis time (1–6 h), proteases were inactivated at a 95°C water bath for 20 min and centrifuged at 9,000 rpm for 15 min. The supernatant was desalted, freeze-dried, and deposited in −20°C. Milt hydrolysate generated by Alcalase showed the highest ACEi activity and referred to TMH.

### Separation process of ACEi peptides from TMH

#### Ultrafiltration

TMH (100.0 mg/mL) was processed with 1, 3.5, and 5 kDa molecular weight (MW) cutoff membranes, and four fractions including TMH-I (<1 kDa), TMH-II (1–3.5 kDa), TMH-III (3.5–5 kDa), and TMH-IV (>5 kDa) were enriched and freeze-dried in vacuum. TMH-I exhibited the maximum ACEi ability among four prepared fractions.

#### Gel permeation chromatography (GPC)

TMH-I solution (5 mL, 50.0 mg/mL) was purified with the Sephadex G-25 column (3.6 × 150 cm) and eluted with phosphate-buffered solution (PBS, 0.2 M) at a flow rate of 0.6 mL/min. The eluate was monitored at 214 nm and collected one tube per 1.8 mL. In consequence, four subfractions (GH-1–GH-4) were isolated from TMH-I, and GH-3 with the maximum ACEi activity was selected for next purification.

#### Reversed-phase high-performance liquid chromatography (RP-HPLC)

GH-3 solution (20 μL, 100.0 μg/mL) was finally separated by RP-HPLC on a Zorbax 300SB-C18 column (4.6 × 250 mm, 5 μm), with a linear gradient of acetonitrile (containing 0.06% TFA) from 0 to 100 % in 0 to 30 min. The eluate with a flow rate of 1.5 mL/min was monitored at 214 nm. At last, 10 ACEi peptides (TP1–TP10) were prepared on their chromatographic peaks.

#### Identification of sequence and MWs of ACEi peptides

The sequences of TP1–TP10 were analyzed using an Applied Biosystems 494 protein sequencer (Perkin Elmer, USA) (22). Edman degradation was performed according to the standard program supplied by Applied Biosystems (Shimazu, Kyoto, Japan). The MWs of TP1–TP10 were determined by using a Q-TOF mass spectrometric device combined with an ESI source (47). Nitrogen was maintained at 40 psi for nebulization and 9 L/min at 350°C for evaporation temperature. The data were collected in the centroid mode from m/z 200 to 2,000.

#### Molecular docking experiment of TP6, TP7, and TP10

This assay of TP6, TP7, and TP10 was performed according to the previous method (54) and commissioned to Shanghai NovoPro Biotechnology Co., Ltd (China). The crystal structures of the human ACE–lisinopril complex (1O8A.pdb) and captopril were acquired from the RCSB PDB Protein Data Bank (PDB code: 1UZF) (https://www.rcsb.org/). The interaction between ACE and MCO was analyzed to determine the position and size of the binding pocket using Chimera software. All non-standard residues in the 1UZF model were deleted, and AutodockTools was used to convert PDB files into PDBQT files (adding Gasteiger charge and setting key distortion). Peptide molecules were converted into a SMILES format by PepSMI tool, 3D models were drawn by Discovery Studio program, and energy minimization was done using steepest descent and conjugate gradient techniques. Molecular docking and free energy calculation were carried out using a flexible docking tool of AutoDock Vina. Finally, the interaction between ACE and peptide molecules was analyzed by Chimera software. According to the binding energy value and scores of TP6, TP7, and TP10, their best ranked docking poses in the active site of ACE were acquired.

### Effects of TP6, TP7, and TP10 on HUVECs

#### Cytotoxic assay

The cytotoxic assay was carried out as per the previous method (54). HUVECs were cultured at a density of 1 × 10^4^ cells/cm^2^ to confluence in DMEM at 37°C in a humidified 5% CO_2_ atmosphere (8). The cytotoxicity of TP1–TP10 on HUVECs was measured using MTT assay (8). In short, HUVECs in 96-well plates at a density of 1 × 10^4^ cells/cm^2^ were separately treated with 20 μL samples at 50 and 200 μM, respectively, and cultured for 24 h. Then, 20 μL MTT solution (5 mg/mL) was put in and incubated for 4 h. In the end, DMSO was joined in each well plate, and the absorbance (A) at 490 nm was determined.


$$\begin{array}{l} \text{Cell viability}\left(\text{\%}\right)=\;\left(A_{\text{sample}}/A_{\text{control}}\right)\;\times \;100. \end{array}$$

#### Determination of nitric oxide (NO) and endothelin-1 (ET-1) production

HUVECs were cultured in 96-well plates at a density of 1 × 10^4^ cells/cm^2^ and treated with Cap (1 μM), NE (0.5 μM), or ACEi peptides (100–200 μM) for 24 h, or incubated with both NE (0.5 μM) and 200 μM ACEi peptides for 24 h. NO and ET-1 contents of HUVECs were determined after 24 h according to the NO and ET-1 assay kits as manufactures' protocol (54).

#### Cytoprotection of TP6, TP7, and TP10 on H_2_O_2_-damaged HUVECs

The cytoprotective assay was carried out using the described methods (55, 56). In short, HUVECs were cultured in a 96-well plate at a density of 1 × 10^4^ cells/cm^2^ for 24 h. Afterward, the supernatant was aspirated, and 20 μL of ACEi peptides (TP6, TP7, and TP10) with the final concentrations of 100 and 200 μM were added to the protection groups, respectively. ACEi peptides (TP6, TP7, and TP10) were removed after 8 h, and H_2_O_2_ with the final concentration of 400 μM was added to the damage and protection groups and incubated for 24 h. GSH was used as the positive control.

The level of ROS was determined on the method described by Cai et al. (55) and expressed as % of blank control; the levels of MDA, SOD, and GSH-Px were measured using assay kits in accordance with the manufacturer' protocols and expressed as U/mg prot.

### Data analysis

All data are expressed as mean ± SD (*n* = 3) and analyzed by SPSS 19.0. The ANOVA test with the Dunnett or Tukey test was employed to analyze the significant difference of samples at different levels (*P* < 0.05, 0.01, or 0.001).

## Results and discussion

### Preparation of milt protein hydrolysate

**Figure 1:**
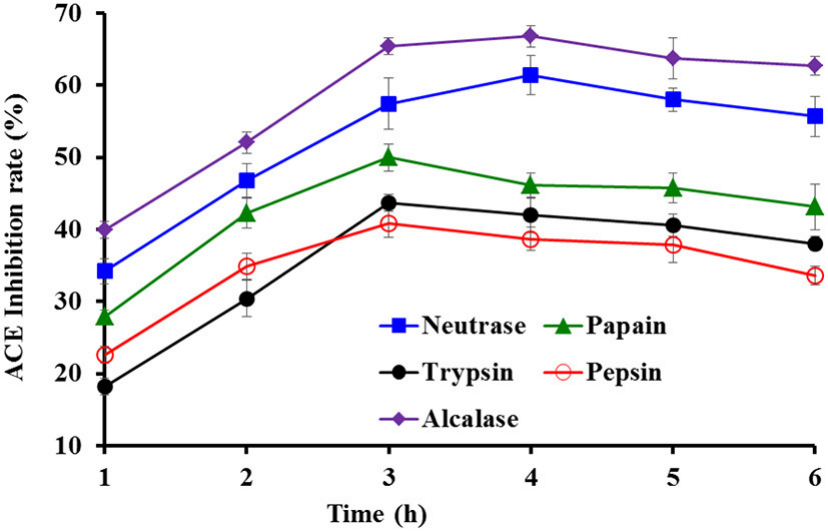
Effects of protease species and hydrolysis time on ACEi activities of protein hydrolysates from skipjack tuna milts at 2.5 mg/ml.

Protein hydrolysates of tuna milts were separately generated using five proteases (Figure 1). The data indicated that the ACEi rates of prepared hydrolysates from tuna milts were significantly affected by protease species and enzymatic time. At the same hydrolysis time, the ACEi rate of Alcalase hydrolysate was markedly higher than that of other four generated hydrolysates (*P* < 0.05). In addition, the ACEi rate of Alcalase hydrolysate produced at 4.0 h was 64.81 ± 2.16%, which was markedly higher than that of hydrolysates produced at other designed time (*P* < 0.05).

Compared with microorganism fermentation and chemical degradation processes, proteolytic hydrolysis method has been widely used because of its advantages of easy control, environmental friendliness, no residual chemical reagents, or no harmful substances (20, 57, 58). The biological activities of protein hydrolysates are closely contacted with the bio-peptide composition, and specificity of enzymes is the key factor affecting those properties (59, 60). Therefore, various proteases and their combinations are designed to produce hydrolysates (20, 61, 62). The results in Figure 1 indicated that Alcalase hydrolysate (TMH) of tuna milts for 4.0 h was most suitable to be selected for the next step of purification.

### Preparing ACEi peptides from TMH

#### Ultrafiltration

**Figure 2:**
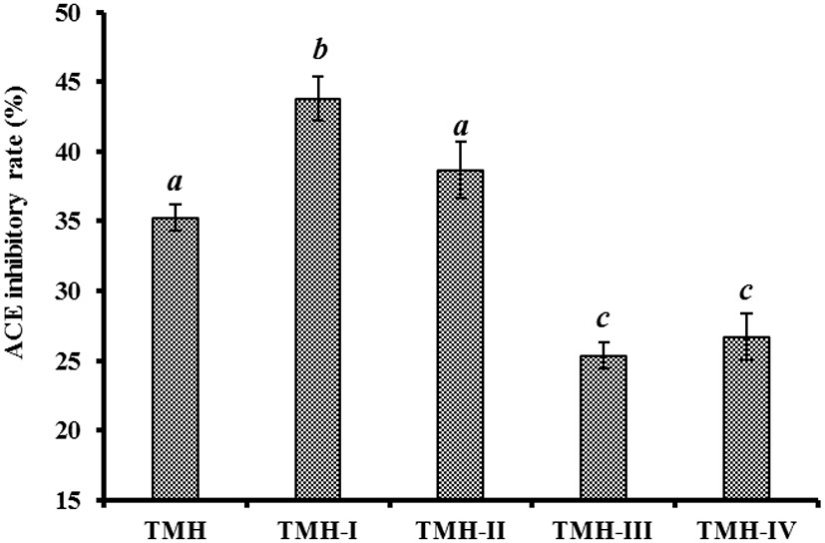
ACEi rates of ultrafiltration peptide fractions (TMH-I to TMH-IV) of protein hydrolysates (TMH) of skipjack tuna milts at a concentration of 1.0 mg/mL. ^a−*c*^Values with same letters indicated no significant difference (*P* > 0.05).

According to Figure 2, the ACEi activity of TMH-I was 43.78 ± 1.56% at 1.0 mg/mL, which was observably higher than that of TMH (35.26 ± 0.95%) and other three ultrafiltration fractions, including TMH-II (38.66 ± 2.04%), TMH-III (25.33 ± 0.95%), and TMH-IV (26.72 ± 1.68%) (*P* < 0.05). Large polypeptides are difficult to get in and combine with the key site of ACE, leading to decreased inhibitory activity (7, 63). Therefore, a short peptide fraction is often isolated from protein hydrolysates by ultrafiltration technology (20, 22, 64). The present results are consistent with those of the literature that the lowest MW peptide fractions from *M. mustelus* (65), Antarctic krill (8), tuna frame (27), *O. kenojei* (9), and *Cyclina sinensis* (66) presented the strongest ACEi activities. As a result, TMH-I with the smallest MW was selected for the next step isolation.

#### GPC of TMH-I

**Figure 3:**
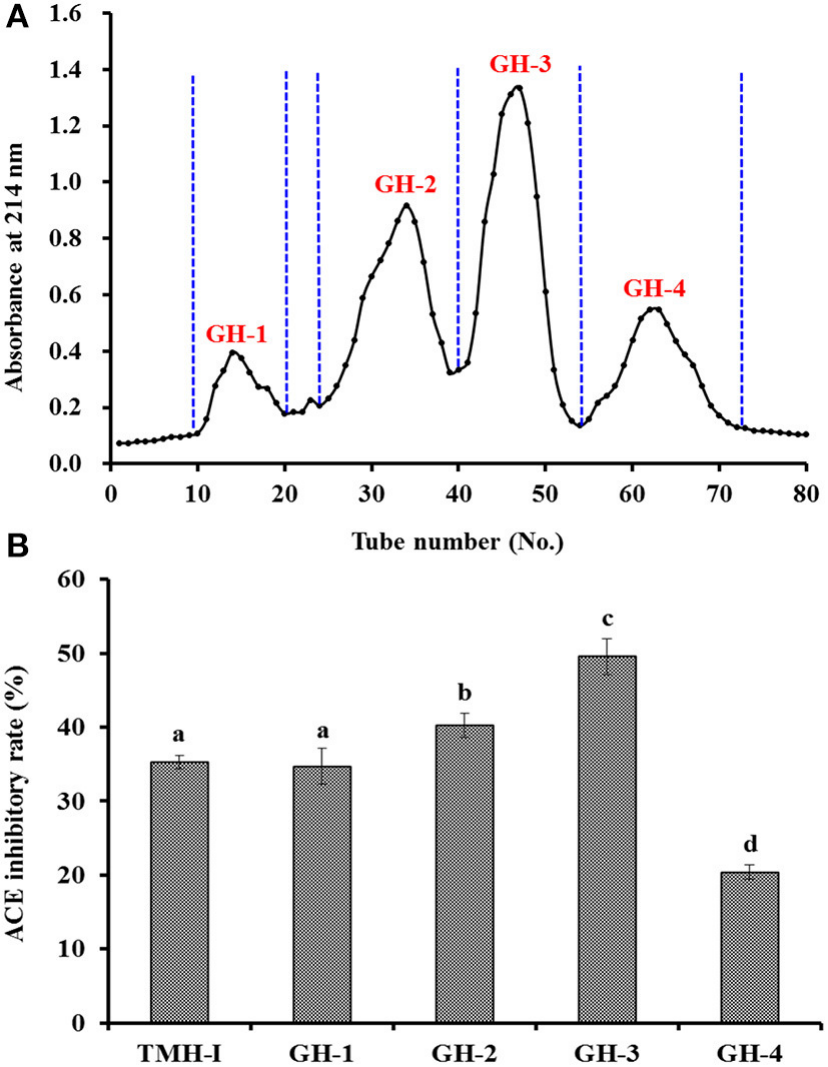
Chromatogram profiles of TMH-I isolated by Sephadex G-25 **(A)** and ACEi rates of prepared subfractions (GH-1–GH-4) from TMH-I at a concentration of 1.0 mg/mL **(B)**. ^a−*d*^Values with the same letters indicated no significant difference (*P* > 0.05).

Figure 3 shows that the ACEi rate of GH-3 at 1.0 mg/mL was 49.52 ± 2.38%, which was far (*P* < 0.05) higher than that of TMH-I (35.26 ± 0.95%) and other three GPC fractions, including GH-1 (34.69 ± 2.41%), GH-2 (40.21 ± 1.65%), and GH-4 (20.36 ± 0.97%). Gel filtration is an effective way to fractionate bioactive molecules in mix ingredients into fractions with particular MW dimensions and is applied frequently for group isolation of protein hydrolysates from different sea foods, such as *C. sinensis* (66), monkfish (*Lophius litulon*) muscle (67), miiuy croaker (22, 62), skipjack tuna byproducts (53–57), red stingray (*Dasyatis akajei*) cartilages (68), and Antarctic krill (8). GH-3 showed the strongest activity, but it does not have the lowest MW. These finding manifested that other factors in addition to MW also significantly affect the ACEi capability of peptides (7, 20, 65).

#### RP-HPLC purification of GH-3

**Figure 4:**
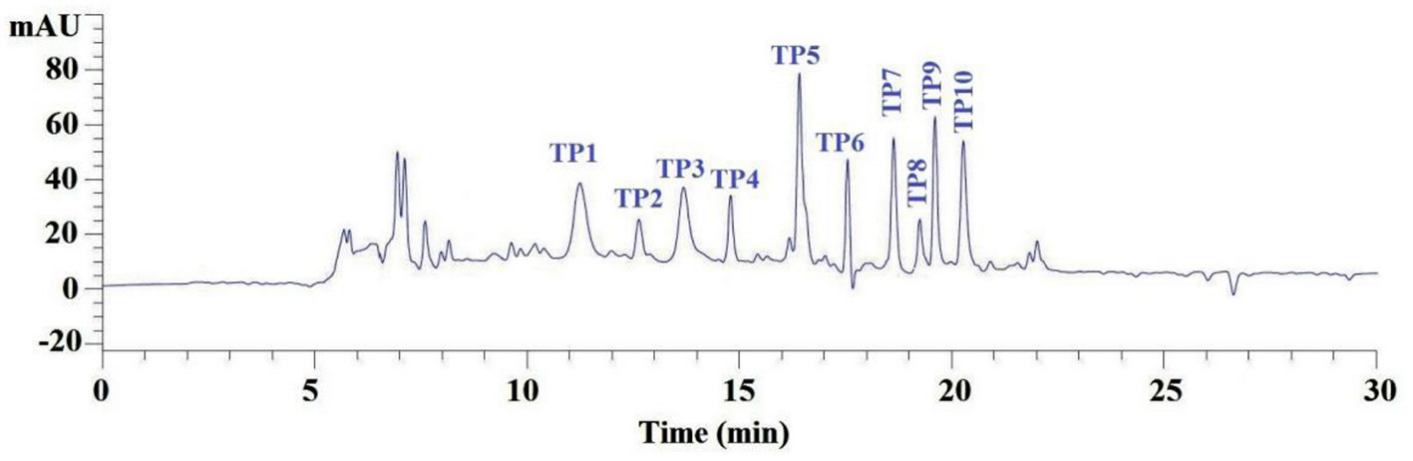
Elution profile of subfraction GH-3 by RP-HPLC using a gradient of acetonitrile containing 0.06% trifluoroacetic acid at 214 nm.

According to the RP-HPLC profiles of GH-3 at 214 nm (Figure 4), 10 ACEi peptides were separately collected on their retention time of 11.24 min (TP1), 12.65 min (TP2), 13.70 min (TP3), 14.76 min (TP4), 16.48 min (TP5), 17.53 min (TP6), 18.64 min (TP7), 19.24 min (TP8), 19.60 min (TP9), and 20.27 min (TP10) (Table 1).

**Table 1:** Amino acid sequences, molecular weights (MWs), and ACEi activity (IC_50_ value) of 10 isolated ACEi peptides (TP1–TP10) from protein hydrolysates of skipjack tuna milts (TMH).

	**Retention time (min)**	**Amino acid sequence**	**Observed MW/theoretical MW (Da)**	**ACEi activity (IC**_50_ **, mg/mL)**
TP1	11.24	Tyr-Asp-Asp (YDD)	411.35/411.36	1.26 ± 0.11^a^
TP2	12.65	Thr-Arg-Glu (TRE)	404.41/404.42	3.73 ± 0.14^b^
TP3	13.70	Arg-Asp-Tyr (RDY)	452.45/452.46	4.35 ± 0.21^c^
TP4	14.76	Thr-Glu-Arg-Met (TERM)	535.60/535.61	1.29 ± 0.09^a^
TP5	16.48	Asp-Arg-Arg-Tyr-Gly (DRRYG)	665.69/665.70	5.74 ± 0.26^d^
TP6	17.53	Ile-Cys-Tyr (ICY)	397.48/397.49	0.48 ± 0.03^e^
TP7	18.64	Leu-Ser-Phe-Arg (LSFR)	521.61/521.61	0.59 ± 0.05^e^
TP8	19.24	Gly-Val-Arg-Phe (GVRF)	477.55/477.56	1.08 ± 0.07 ^a^
TP9	19.60	Lys-Leu-Tyr-Ala-Leu-Phe (KLYALF)	753.91/753.93	3.41 ± 0.19^f^
TP10	20.27	Ile-Tyr-Ser-Pro (IYSP)	478.53/478.54	0.76 ± 0.04^g^

^a−g^*Values with same letters indicated no significant difference (P > 0.05)*.

#### Peptide sequence and MW determination

**Figure 5:**
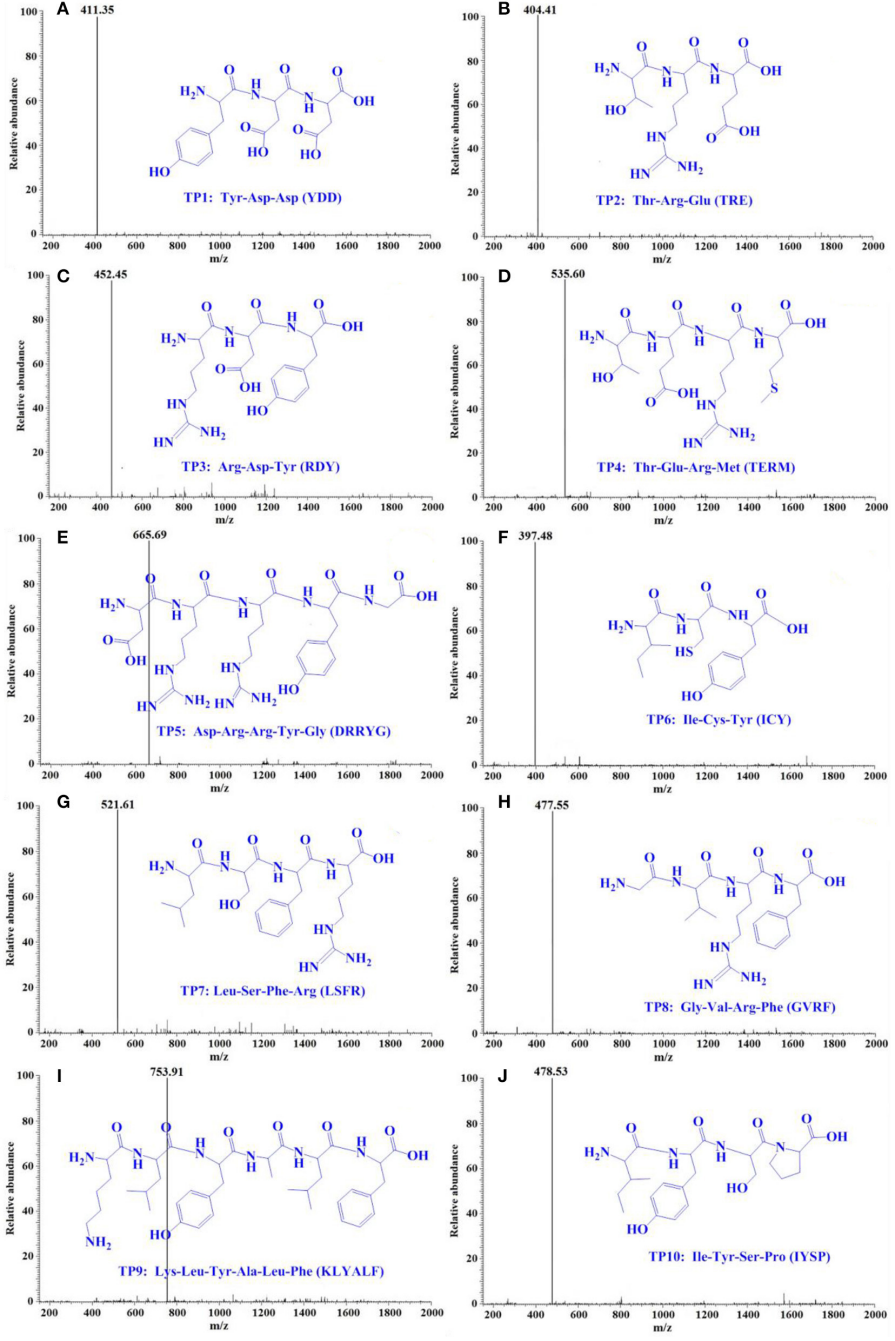
Mass spectrogram of 10 ACEi peptides (TP1–TP10) from protein hydrolysate of skipjack tuna milts (TMH). **(A)** TP1, **(B)** TP2, **(C)** TP3, **(D)** TP4, **(E)** TP5, **(F)** TP6, **(G)** TP7, **(H)** TP8, **(I)** TP9, and **(J)** TP10.

Using a protein/peptide sequencer, peptide sequences of TP1–TP10 were identified as Tyr-Asp-Asp (YDD, TP1), Thr-Arg-Glu (TRE, TP2), Arg-Asp-Tyr (RDY, TP3), Thr-Glu-Arg-Met (TERM, TP4), Asp-Arg-Arg-Tyr-Gly (DRRYG, TP5), Ile-Cys-Tyr (ICY, TP6), Leu-Ser-Phe-Arg (LSFR, TP7), Gly-Val-Arg-Phe (GVRF, TP8), Lys-Leu-Tyr-Ala-Leu-Phe (KLYALF, TP9), and Ile-Tyr-Ser-Pro (IYSP, TP10). The MWs of the 10 ACEi peptides (TP1–TP10) were determined as 411.35, 404.41, 452.45, 535.60, 665.69, 397.48, 521.61, 477.55, 753.91, and 478.53 Da, respectively (Figure 5), which agreed well with their theoretical MWs (Table 1).

#### ACEi activity and molecular docking analysis

The IC_50_ values of TP6, TP7, and TP10 on ACE were 0.48 ± 0.03, 0.59 ± 0.05, and 0.76 ± 0.04 mg/mL (Table 1), respectively, which were markedly lower than those of other seven ACEi peptides (*P* < 0.05). Moreover, the IC_50_ values of TP6, TP7, and TP10 were less than those of ACEi peptides from *Salmo salar* (YP: 1.54 mg/mL) (69), Antarctic krill (VD: 5.61 mg/mL; FRKE: 6.97 mg/mL) (8), *Ctenopharyngodon idella* (VAP: 1.71 mg/mL) (1), skate (LGPLGHQ: 4.22 mg/mL) (29), *Katsuwonus pelamis* (MLVFAV: 2.36 mg/mL) (70), and stone fish (EHPVL: 1.68 mg/mL) (2). The present results demonstrated that TP1–TP10, especially TP6, TP7, and TP10, had prominent ACEi ability and could be used as function components in reducing blood pressure products.

**Figure 6:**
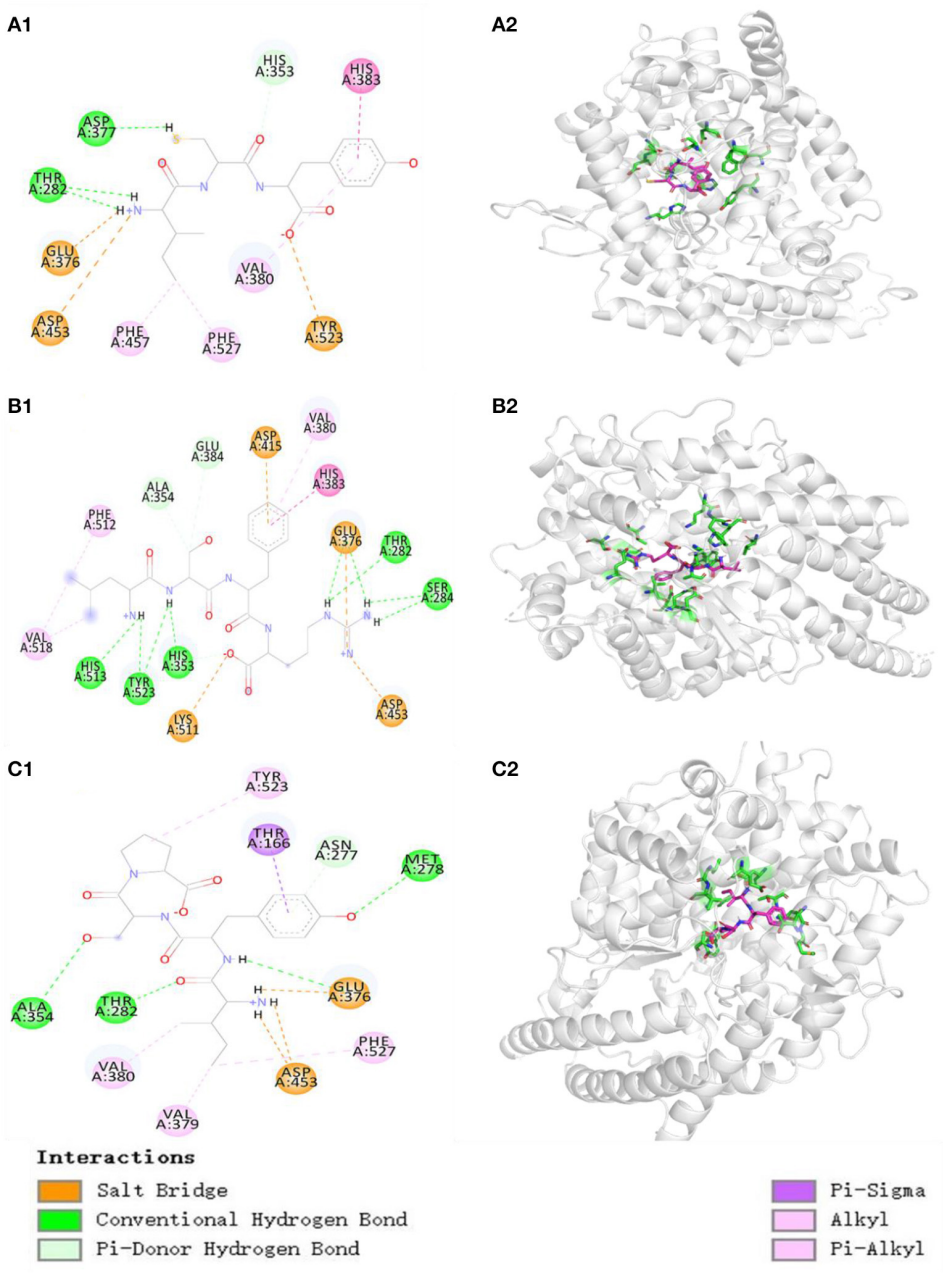
Molecular docking results of TP6, TP7, and TP10 with ACE. **(A1)** 2-D details of ACE and TP6 interaction. **(A2)** 3-D interaction details for TP6; **(B1)** 2-D details of ACE and TP7 interaction. **(B2)** 3-D interaction details for TP7; **(C1)** 2-D details of ACE and TP10 interaction. **(C2)** 3-D interaction details for TP10.

The molecular docking experiment served to illustrate the action mechanisms of TP6, TP7, and TP10 in inhibiting ACE (Figure 6). Figure 6A proves that TP6 (ICY) formed hydrogen bonds with His353, Asp377, and Thr282 residues of ACE, of which TP6 (ICY) formed hydrogen bonds with the active pocket of S2 (His353). In addition, TP6 (ICY) interacted with His383, Phe457, Phe527, and Val380 residues of ACE through a hydrophobic effect, and contacted with Asp453, Glu376, and Tyr523 residues of ACE through an electrostatic force. Figure 6B proves that TP7 (LSFR) built hydrogen bonds with Ala354, Glu384, Thr282, Ser284, His353, Tyr523, and His513 (S2) residues of ACE, among which TP7 (LSFR) formed hydrogen bonds with active pockets of S1 (Ala354, Glu384, and Tyr523) and S2 (His353 and His513). In addition, TP7 (LSFR) contacted with Val380, His383, Phe512, and Val518 residues of ACE through a hydrophobic effect and contacted with Glu376, Asp415, Asp453, and Lys511 residues of ACE through an electrostatic force. Figure 6C reveals that TP10 (IYSP) built hydrogen bonds with Ala354, Thr282, Met278, and Asn277 residues of ACE, among which TP10 (IYSP) formed hydrogen bonds with the active pocket of S1 (Ala354). In addition, TP10 (IYSP) interacted with Val380, Val379, Phe527, Thr166, and Tyr523 residues of ACE through a hydrophobic effect and contacted with Glu376 and Asp453 residues of ACE through electrostatic force. The experiment illuminated that TP6, TP7, and TP10 exhibited strong ACEi ability attributing to effective binding with the key sites of ACE by hydrogen bonding, electrostatic force, and hydrophobic interaction. In addition, the activity of TP6, TP7, and TP10 was correlated with the interaction with S1 and S2 pockets of ACE.

In addition, the affinity of TP6, TP7, and TP10 with ACE was−7.0,−8.5, and−8.3 kcal/mol, which were close to those of SP (5.7 kcal/mol), VDRYF (9.7 kcal/mol), and YSK (−7.9 kcal/mol) from tuna muscle (54) and rice bran (72).

Molecular size significantly impacts the affinity between peptides and ACE because large peptides cannot pass through the narrow binding channel of ACE (7, 71). For example, VPP and IPP could conveniently pass through the ACE channel and combine with Zn^2+^, but 7–11 peptides, including TTMYPGIA, AVVPPSDKM, GPAGPRGPAG, and ALPMHIR, revealed weak affinity with ACE (34). In the experiment, TP6, TP7, and TP10 are tripeptides or tetrapeptides, and their small MWs increase their chances of getting close to the binding channel of ACE, and this was proved by their affinities with ACE (−7.0, −8.5, and −8.3 kcal/mol for TP6, TP7, and TP10, respectively).

Amino acids, especially the C- and N-terminal amino acids, are crucial to the ACEi activity of oligopeptides (5, 7). The aromatic (Tyr and Phe) and branched-chain (Leu and Ile) amino acids were the key residues in the C terminus of oligopeptides (72, 73). Amino acid residues containing a positive charge, such as Lys and Arg, the C terminus could be conducive to heighten the ACEi activity (74). Hayes et al. demonstrated that hydrophobic amino acids are favorable to bind to the key site of ACE (75). The Pro residue was the critical residue in the C terminus of KDEDTEEVP, ADVFNPR, LPILR, VGLYP, and VIEPR. Moreover, the Pro residue was proved that it could improve the resistant ability of oligopeptides against the digestion of the gastrointestinal tract (5, 73). Therefore, Tyr, Arg, and Pro at the C terminus of TP6, TP7, and TP10 are specially vital for their ACEi activity, and this was also proved by the results in Figure 6.

In addition, the role of N-terminal amino acids is also emphasized and discussed. Moayedi et al. reported that branched aliphatic Val, Leu, and Ile residues at its N terminus could exert strong inhibitory ability against ACE (76). Auwal et al. reported a similar result that branched aliphatic amino acids in the N terminus could improve the ACEi ability of peptides (2). Therefore, the Leu residue at N-terminal TP7 and Ile residue at N-terminal TP6 and TP10 play vital effects on their ACEi ability.

### Effects of TP6, TP7, and TP10 on HUVECs

#### Effects of TP6, TP7, and TP10 on cell viability

**Figure 7:**
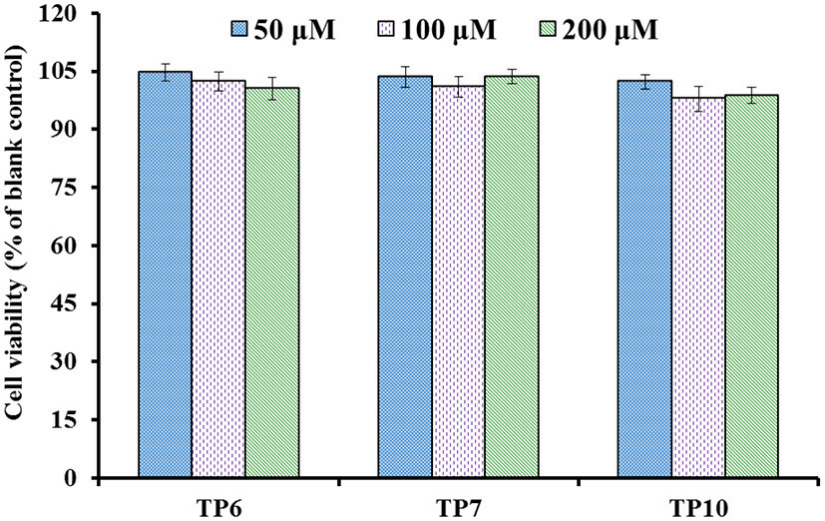
Cell viability of HUVECs treated with TP6, TP7, and TP10 for 24 h, respectively.

The influences of TP6, TP7, and TP10 on the viability of HUVECs at 50–200 μM are presented in Figure 7, and their cell viability ranged from 98.76 ± 2.09% to 104.65 ± 2.19%. These data indicated that TP6, TP7, and TP10 have no significant toxicity to HUVECs.

Endothelial cells (HUVECs) constitute the inner cellular lining of blood vessels and play an important role in serial physiopathological processes, for instance, infection, repair in trauma, angiogenesis, and atherosis (5, 7). Then, HUVECs are currently considered to be one of the model cells in curing the disease of the cardiovascular system (8, 54, 77). The cell proliferation and death generally keeps an appropriate balance in normal tissues, and the active substances with strong inhibiting ability on cell proliferation illustrate their possible cytotoxicity risk to the life body and are deemed to be inadequate to develop healthy products with antitumor functions (7, 78). These current findings proved that TP6, TP7, and TP10 were do not render obvious toxicity to endothelial cells and should suite to developing anti-blood pressure health products.

#### Effects of TP6, TP7, and TP10 on NO production and ET-1 secretion

**Figure 8:**
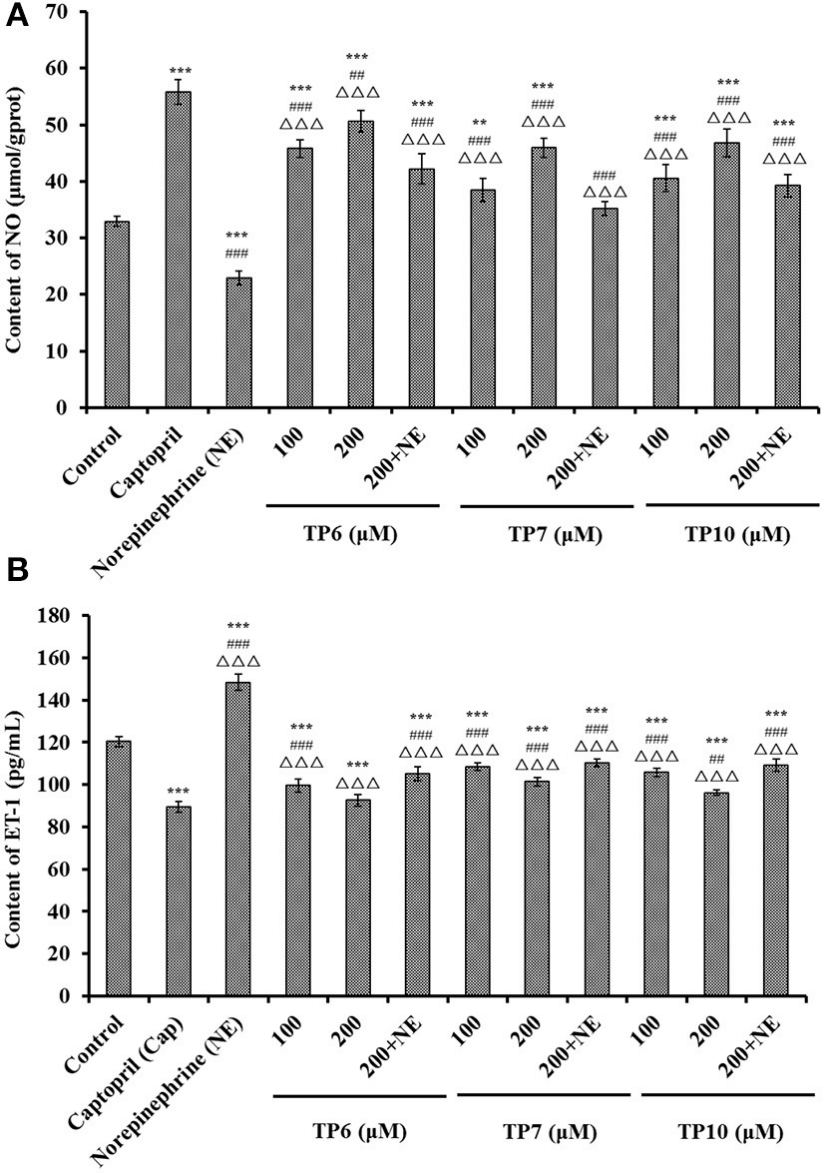
Contents of nitric oxide (NO) **(A)** and endothelin-1 (ET-1) **(B)** of HUVECs treated with TP6, TP7, and TP10 for 24 h, respectively. The cell group treated with captopril (Cap) was designed as the positive control. ***P < 0.001 vs. control; ^###^*P* < 0.001 and ^##^*P* < 0.01 vs. captopril; ^ΔΔΔ^*P* < 0.001 vs. norepinephrine (NE).

Figure 8A shows that the NO levels in HUVECs incubated with TP6, TP7, and TP10 were significantly increased in comparison with the control group (*P* < 0.001), and the NO levels of TP6, TP7, and TP10 groups increased to 50.63 ± 1.95, 45.91 ± 1.68, and 46.78 ± 2.47 μmol/gprot at 200 μM. In addition, NE could markedly downregulate the level of NO (22.91 ± 1.26 μmol/gprot) in comparison with the control group (*P* < 0.001), but the NO content decreased by NE was separately compensated to 42.21 ± 2.66, 35.18 ± 1.2, and 39.27 ± 1.96μmol/gprot in TP6, TP7, and TP10 groups at 200 μM (*P* < 0.001).

Figure 8B indicates that TP6, TP7, and TP10 could dramatically decrease the ET-1 secretion of HUVECs (*P* < 0.001), and the ET-1 levels of TP6, TP7, and TP10 groups reduced to 92.57 ± 2.68, 101.49 ± 2.05, and 96.15 ± 1.35 pg/mL at 200 μM. Compared to the control group, NE could prominently enhance the ET-1 secretion (148.35 ± 3.87 pg/mL) (*P* < 0.001), but this negative effect on ET-1 secretion was partially supplemented by TP6, TP7, and TP10 treatment and lowered to 105.18 ± 3.29, 110.36 ± 1.76, and 109.12 ± 2.95 pg/mL at 200 μM (*P* < 0.001).

In pathologic situations, NO deficiency will give rise to the risks of cardiovascular diseases, and improving the production of endothelial NO represents a good therapeutic approach for atherosclerosis (8, 54). Therefore, some ACEi peptides, such as KYIPIQ (79), WF (8), GRVSNCAA, TYLPVH (80), SP (54), and MKKS and LPRS (81), play their hypotensive activity by enhancing the production of NO in HUVECs. As a functional factor similar to Ang II, ET-1 can lead to endothelial dysfunction correlated with coronary heart disease and hypertension (8). VVLYK from palm kernel expeller could dose-dependently inhibit the secretion of intracellular ET-1 in EA.hy926 cells (73). GRVSNCAA and TYLPVH from *Ruditapes philippinarum* lowered blood pressure by markedly lowering ET-1 generation (80). In addition, oligopeptides of SP, YRK, MKKS, FQK, FAS, and LPRS from tuna muscles and Antarctic krill displayed a similar function of decreasing the ET-1 level (8, 54, 81). According to this finding, ACEi peptides of TP6, TP7, and TP10 prominently promote NO production while restricting ET-1 secretion in HUVECs. Moreover, TP6, TP7, and TP10 can reverse the negative effect of NE upon NO- and ET-1-producing processes in HUVECs.

### Antioxidant functions of TP6, TP7, and TP10 on H_2_O_2_-damaged HUVECs

#### Influence of TP6, TP7, and TP10 on viability of H_2_O_2_-damaged HUVECs

**Figure 9:**
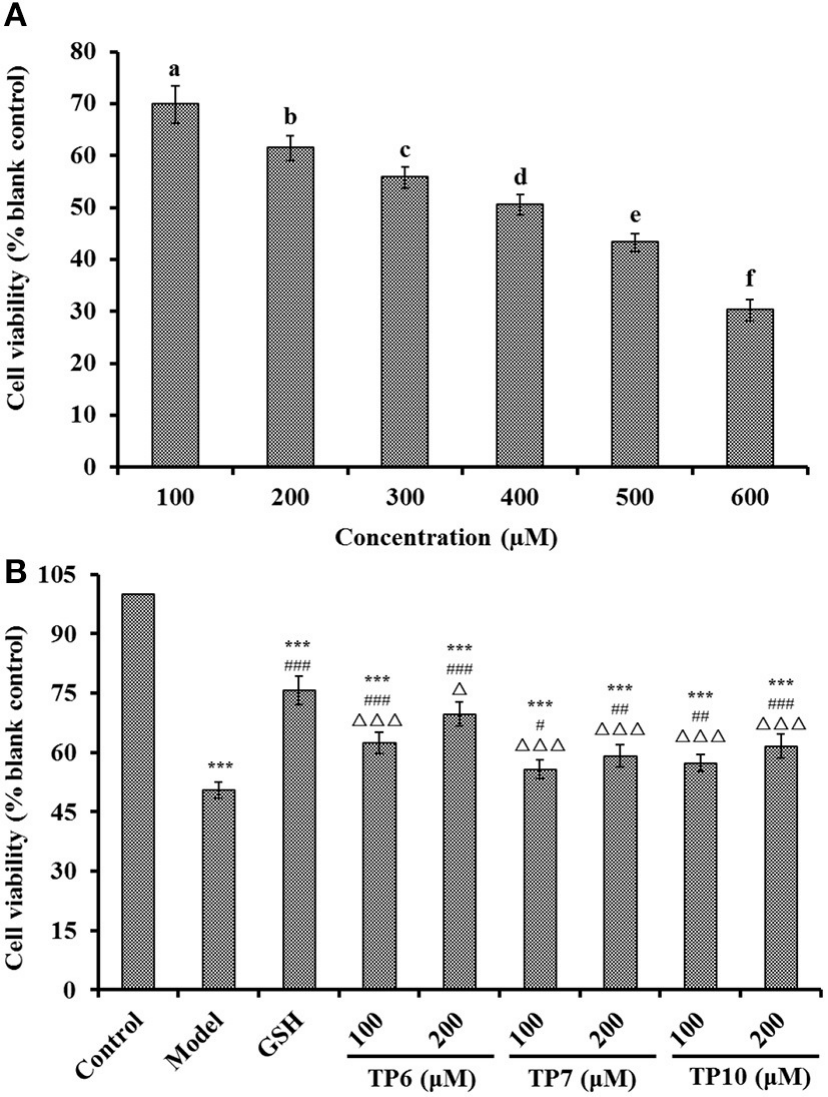
Effects on the viability of different H_2_O_2_ concentration (100–600 μM)-treated HUVECs **(A)** and TP6-, TP7-, and TP10-treated H_2_O_2_-damaged HUVECs **(B)**. **(A)** ^a−*f*^Values with same letters indicate no significant difference (*P* > 0.05); **(B)** ***P < 0.001 vs. control; ^#^*P* < 0.05, ^##^*P* < 0.01, and ^###^*P* < 0.001 vs. model; ^Δ^*P* < 0.05 and ^ΔΔΔ^*P* < 0.001 vs. GSH.

Figure 9A indicates that H_2_O_2_ concentration from 100 to 600 μM had a significant effect on the viability of HUVECs (*P* < 0.05). The literature indicates that the concentration of H_2_O_2_ induced the cell viability of about 50%, which is optimal for establishing an oxidative damage cell model (78). Therefore, the H_2_O_2_ concentration of 400 μM induced the cell viability of 50.48 ± 1.96% of the blank group, which was applied to establish the cell model of oxidative damage. Oxidative stress can cause the excessive accumulation of ROS, which results in damage to HUVECs and further leads to the injury to vascular barrier function, the occurrence of atherosclerosis, high blood pressure, and other cardiovascular diseases (7, 20). Thus, H_2_O_2_-induced HUVECs are preferably applied to explore cellular protective mechanisms of ACEi peptides.

Figure 9B presents the cytoprotective effects of TP6, TP7, and TP10 on the H_2_O_2_-damaged HUVECs at 100 and 200 μM. TP6, TP7, and TP10 showed the significantly protective effects on the H_2_O_2_-damaged HUVECs in a dose-dependent fashion, and the cell viabilities of TP6, TP7, and TP10 groups at 200 μM were increased to 69.76 ± 3.06, 59.15 ± 2.81, and 61.58 ± 3.04%, respectively, which were significantly higher than those of model groups (50.48 ± 1.96%) (*P* < 0.01 or 0.001). However, the cell viability of TP6, TP7, and TP10 groups was inferior to that of the GSH group (75.69 ± 3.52%) (*P* < 0.05 or 0.001). Then, TP6, TP7, and TP10 could dramatically increase cell viability and give a strong protection to H_2_O_2_-induced HUVECs. Zheng et al. found that VIEPR and ADVFNPR from oil palm kernel expeller could exert an antihypertensive effect through scavenging excessive ROS and protect vascular endothelial cells from excessive ROS-induced damage (73). Umami peptides of CC, CCNK, and HCHT could dose-dependently increase the NO concentration and decrease the ET-1 content in HUVECs. Moreover, CC, CCNK, and HCHT showed cytoprotective effects by reducing the ROS content (82). Therefore, TP6, TP7, and TP10 showed a similarly cytoprotective effect on HUVECs with those reported ACEi peptides.

#### Influences of TP6, TP7, and TP10 on ROS, MDA, and antioxidases (SOD and GSH-Px) in H_2_O_2_-damaged HUVECs

**Figure 10:**
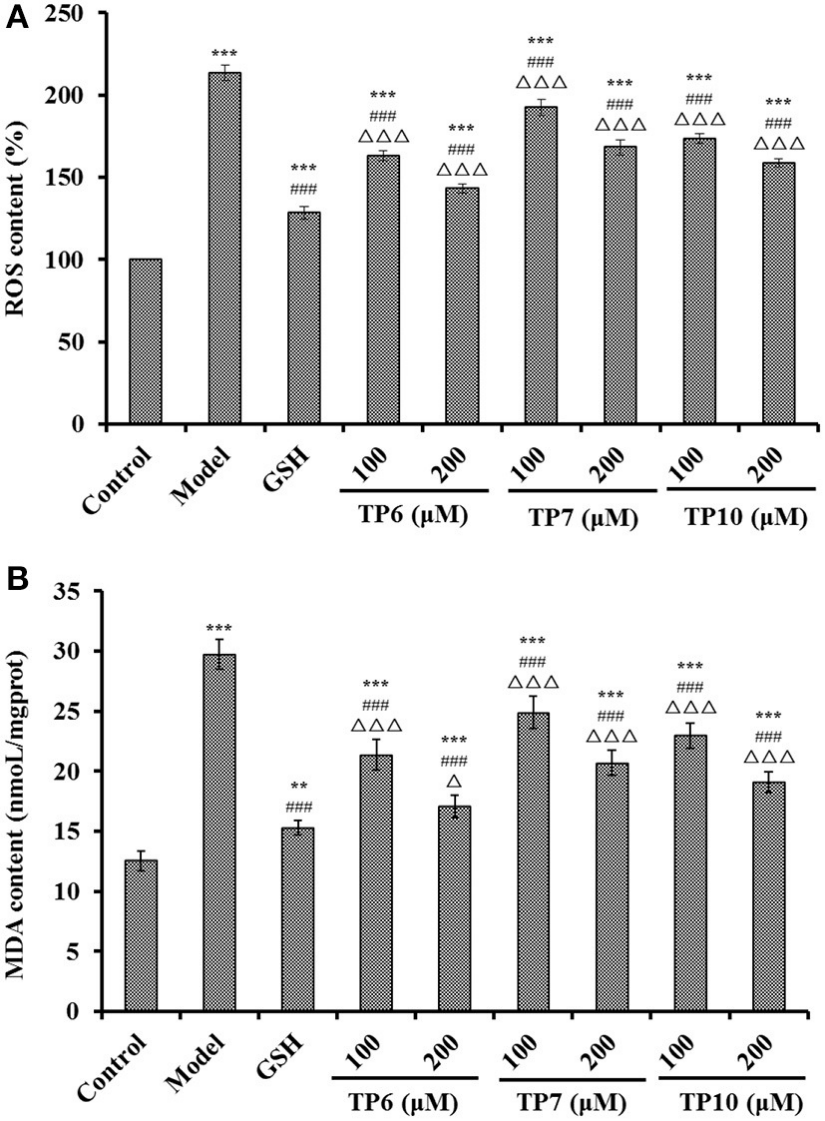
Effects of TP6, TP7, and TP10 on the ROS **(A)** and MDA **(B)** levels of H_2_O_2_-damaged HUVECs. ***P < 0.001 and **P < 0.01 vs. control; ^###^*P* < 0.001 vs. model; ^Δ^*P* < 0.05 and ^ΔΔΔ^*P* < 0.001 vs. GSH.

Figure 10A indicates that the ROS levels were markedly lowered after pretreating with TP6, TP7, and TP10 compared with the model group (213.54 ± 4.62%) (*P* < 0.001). At 200 μM, the ROS levels of TP6, TP7, and TP10 groups were observably dropped to 143.29 ± 2.66, 168.37 ± 4.68, and 158.78 ± 2.59 of the blank control group, respectively. In addition, TP6 showed the strongest ability on scavenging ROS among TP6, TP7, and TP10 groups.

Figure 10B reveals that the MDA levels were markedly lowered after pretreating with TP6, TP7, and TP10 compared with the model group (29.72 ± 1.23 nmol/mg prot) (*P* < 0.001). At 200 μM, the MDA levels of TP6, TP7, and TP10 groups were dramatically decreased to 17.08 ± 0.96, 20.68 ± 1.06, and 19.11 ± 0.88 nmol/mg prot, respectively. TP6 showed the strongest ability on decreasing the MDA content among TP6, TP7, and TP10 groups, but its ability was still inferior to that of GSH (15.28 ± 0.61 nmol/mg prot).

**Figure 11:**
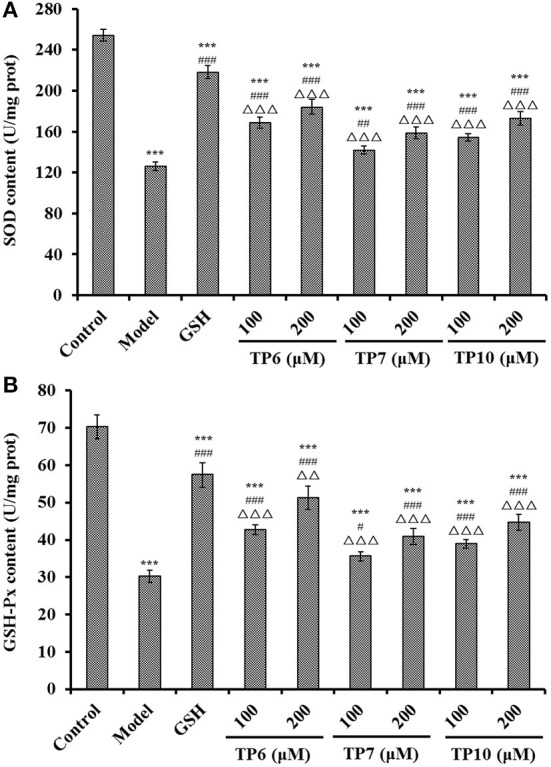
Effects of TP6, TP7, and TP10 on the SOD **(A)** and GSH-Px **(B)** levels of H_2_O_2_-damaged HUVECs. All values are means ± SD (*n* = 3). ***P < 0.001 and **P < 0.01 vs. control; ^#^*P* < 0.05, ^*##*^*P* < 0.01, and ^###^*P* < 0.001 vs. model; ^ΔΔ^*P* < 0.01 and ^ΔΔΔ^*P* < 0.001 vs. GSH.

Figure 11A shows the activities of SOD and GSH-Px incubated with TP6, TP7, and TP10 at 100 and 200 μM were gradually increased. At the concentrations of 100 and 200 μM, the SOD levels in the TP6 group were 168.77 ± 5.32 and 184.06 ± 7.19 U/mg prot; the activities in TP7 groups were 141.79 ± 3.96 and 158.79 ± 5.93 U/mg prot; and the activities in TP10 groups were 154.35 ± 3.68 and 172.93 ± 6.58 U/mg prot. Moreover, the SOD activities in TP6, TP7, and TP10 groups were markedly higher than those (119.31 ± 9.48 U/mg prot) of the model group (*P* < 0.001).

The changes of GSH-Px levels showed the same trend with the levels of SOD (Figure 11B). At 100 and 200 μM, the GSH-Px levels in the TP6 group were 42.69 ± 1.32 and 51.28 ± 3.16 U/mg prot; the activities in the TP7 group were 35.61 ± 1.23 and 40.93 ± 2.15 U/mg prot; and the activities in the TP10 group were 38.91 ± 1.17 and 44.69 ± 2.09 U/mg prot. The GSH-Px activity of peptide groups were observably higher than that of the model group (30.24 ± 1.61 U/mg prot) (*P* < 0.05 or 0.001).

For maintaining the optimal healthy state of cells, endogenous antioxidant defense systems can timely and efficiently get rid of excessive ROS (78, 83, 84). Also, MDA is a key peroxidation product of the cell membrane lipid and serves as a well-known indicator for estimating the oxidative damage degree (85, 86). Collagen peptides of GASGPMGPR and GLPGPM from yak bones could prominently lower the accumulations of ROS and MDA by strengthening the levels of SOD and CAT in worms (87). FPYLRH, FWKVV, and FMPLH could dose-dependently enhance the levels of SOD and GSH-Px to weaken the damage to DNA and the contents of ROS and MDA in H_2_O_2_-induced HUVECs (55, 78). Peptides from hazelnut byproduct can protect HUVECs against oxidant damage induced by angiotensin II by upregulating the activity of SOD and HO-1 to control ROS generation (88). LKPGN and LQP from Antarctic krill hydrolysate could enhance the activity of SOD and GSH-Px to eliminate superfluous ROS, which further reduces DNA damage and MDA content in H_2_O_2_-induced Chang liver cells (56). Moreover, the antioxidant mechanisms of GPA (89), KVLPVPEK, APKGVQGPNG (90), ICRD, and LCGEC (51) indicated that they could activate the Nrf2 pathway in the oxidative damage cell model to induce the overexpression of GSH-Px, heme oxygenase-1 (HO-1), and SOD to decrease the oxidative damage of ROS. The current findings demonstrated that the protective activities to H_2_O_2_-damaged HUVECs of TP6, TP7, and TP10 were similar to those of previous reported peptides, and the mechanism should be related to activating the Nrf2 pathway to improve antioxidase levels.

## Conclusion

In conclusion, 10 novel ACEi peptides were isolated from the protein hydrolysate of skipjack tuna milts and identified as YDD, TRE, RDY, TERM, DRRYG, ICY, LSFR, GVRF, KLYALF, and IYSP, respectively. Among them, ICY, LSFR, and IYSP displayed noticeable hypotensive activity by inhibiting ACE activity, increasing NO production and decreasing ET-1 secretion in HUVECs, and protecting HUVECs from H_2_O_2_-induced oxidative damage. Moreover, ICY, LSFR, and IYSP exhibited significant ACEi activity attributing to their effective interaction with the active sites of ACE by hydrogen bonding, electrostatic force, and hydrophobic interaction. Therefore, this study not only develops technical support for utilizing skipjack tuna milts to produce novel ACEi peptides but also contributes to dispose the environmental pollution problems of tuna byproducts. More importantly, 10 novel ACEi peptides, especially ICY, LSFR, and IYSP, might be used as natural functional ingredients for developing noticeable hypotensive products. However, investigating the antihypertensive activities of ICY, LSFR, and IYSP in mouse models should be explored in future studies, which will provide better insights into their potential in the management of hypertension.

## Data availability statement

The raw data supporting the conclusions of this article will be made available by the authors, without undue reservation.

## Author contributions

S-KS, S-LZ, and C-FC: data curation, methodology, and formal analysis. H-YL: methodology, conceptualization, supervision, funding acquisition, and writing—review and editing. BW: supervision, funding acquisition, and writing—review and editing. All authors have read and agreed to the published version of the manuscript, contributed to the article, and approved the submitted version.

## Funding

This work was funded by the National Natural Science Foundation of China (No. 82073764) and Ten-Thousand Talents Plan of Zhejiang Province (No. 2019R52026).

## Conflicts of interest

The authors declare that the research was conducted in the absence of any commercial or financial relationships that could be construed as a potential conflict of interest.

## Publisher's note

All claims expressed in this article are solely those of the authors and do not necessarily represent those of their affiliated organizations, or those of the publisher, the editors and the reviewers. Any product that may be evaluated in this article, or claim that may be made by its manufacturer, is not guaranteed or endorsed by the publisher.
